# Psychometric Evaluation of Social Cognition and Behavior Measures in Children and Adolescents with Down Syndrome

**DOI:** 10.3390/brainsci11070836

**Published:** 2021-06-24

**Authors:** Emily K. Schworer, Emily K. Hoffman, Anna J. Esbensen

**Affiliations:** 1 Division of Developmental and Behavioral Pediatrics, Cincinnati Children’s Hospital Medical Center, Cincinnati, OH 45229, USA; Emily.Hoffman1@cchmc.org (E.K.H.); Anna.Esbensen@cchmc.org (A.J.E.); 2 Department of Pediatrics, University of Cincinnati College of Medicine, Cincinnati, OH 45267, USA

**Keywords:** Down syndrome, social cognition, social behavior, measurement, children

## Abstract

Individuals with Down syndrome (DS) are often described as socially engaged; however, challenges with social cognition, expressive language, and social interaction are also common in DS and are prospective outcomes of interest for clinical trials. The current study evaluates the psychometric properties of standardized measurements of social cognition and social behavior for potential use as outcome measures for children and adolescents with DS. Seventy-three youth ages 6 to 17 years old (M = 12.67, SD = 3.16) with DS were assessed on social cognition subtests of a neuropsychological assessment at two time points. Caregivers also completed a parent-report measure of social behavior. Measures were evaluated for feasibility, test-retest reliability, practice effects, convergent validity, and associations with broader developmental domains (i.e., age, cognition, and language). All social cognition and behavior measures met criteria for a portion of the psychometric indices evaluated, yet feasibility limitations were identified for the Developmental Neuropsychological Assessment, Second Edition (NEPSY-II) Affect Recognition subtest, and the NEPSY-II Theory of Mind subtest had problematic floor effects for percentile ranks. The Social Responsiveness Scale, Second Edition (SRS-2; T-scores) had high feasibility, moderate to excellent test-retest reliability, and no practice effects, suggesting this measure could be appropriate for use in clinical trials involving youth with DS.

## 1. Introduction

Individuals with Down syndrome (DS) are commonly described as socially engaged [1] and as having relatively strong nonverbal social functioning in early childhood [2]. Nevertheless, individuals with DS also experience challenges with core aspects of social relatedness including social cognition, expressive language, and social interaction [3,4,5]. Social challenges are further evident in rates of co-occurring autism spectrum disorder (ASD) in DS. Recent studies show that approximately 15–18% of children with DS also have an ASD diagnosis, which is markedly higher than the 1% reported in the general population [6,7]. Children with DS, with and without co-occurring ASD, experience social challenges that impede interactions with peers [5,8]. These social difficulties lead to greater potential for social isolation that, in turn, impacts mental health outcomes for this population [9], making social cognition and other social skills potential targets for intervention.

Social cognition is defined as the understanding of other’s intentions, emotions, and behaviors [9,10,11]. This includes concepts such as theory of mind, which is the ability to reason about another’s point of view, and affect recognition, the ability to identify emotions in others. Social cognition requires individuals to process and interpret social cues, and these skills impact the selection of social responses and subsequent quality of interactions with others in social contexts [12]. In children with ASD, specific connections have been made between social information processing and social behavior [12,13]. In children with DS, theory of mind performance is a greater relative challenge compared with children with other neurogenetic syndromes and intellectual disabilities, and their performance falls below their overall nonverbal cognitive abilities [14,15]. Affect and emotion recognition is also an area of challenge in DS in comparison with children with typical development matched on cognitive or receptive language level [16,17]. Studies using the Social Responsiveness Scale, Second Edition (SRS-2) [18] describe social communication and interactions in individuals with DS at low risk for ASD and show that these individuals have relative strengths in social motivation and challenges with social cognition, communication, and awareness [5,19]. For a review of social cognition development in DS, see [20].

A variety of measures have been used in past social cognition research in DS. Most of these measures are laboratory-based and include false belief tasks involving the location of objects [15,21,22] or the content of a container [15], appearance reality tasks [15,22], and emotion-matching tasks [16,17,23]. Although the majority of research on social cognition is completed with toddlers and preschool-aged children [9], these measures are used in the assessment of older children, adolescents, and young adults with DS [14,15]. Beyond the use of laboratory-based measures, standardized clinical assessments of social cognition have been used successfully to describe performance in other clinical populations such as ASD and Attention-Deficit/Hyperactivity Disorder (ADHD) [24,25]. However, standardized clinical assessments have yet to be evaluated to assess social cognition in DS. Another measure used to assess social cognition, among other social behaviors, is the SRS-2, and previous work supports its utility in 6- to 21-year-olds with DS [5,19]. A benefit of using the SRS-2 is that the parent reports on the child’s typical social behavior without the child having to do more intensive in-person assessments. The SRS-2 is reported to have high internal consistency and concurrent validity with other ASD screeners among children with DS [19]; however, broader examination of test-retest reliability, practice effects, and convergent validity with direct assessments of social cognition has yet to be studied.

As we learn more about the social phenotype of children with DS and DS+ASD and as social challenges are better characterized [8,19], additional socially focused interventions tailored to children with DS will be needed. Pilot interventions targeting theory of mind skills have recently been completed with children and adolescents with DS [26] and suggest that these skills can be improved with targeted behavioral intervention. Because of the prospective growth of studies focused on social cognition and interaction in DS, a necessary first step to intervention studies is to validate social cognition and social behavior measures for this population.

Further, the priority to evaluate outcome measures for interventions and clinical trials in DS was expressed by the 2015 National Institutes of Health Down Syndrome Outcome Measure working group [27,28]. A summary from this working group identified no direct assessments of social cognition with evidence for use in DS but did state that the SRS-2 showed promise based on the sensitivity of the measure to detect ASD symptoms in DS [19,28]. Social cognition measures have been psychometrically evaluated in the general population [29,30,31]; however, continued efforts are needed to determine appropriate measures for DS. Psychometrically evaluating social cognition and social behavior measures in DS will ensure that assessments of these domains are suitable for children with DS and that there are no unintended floor effects due to the behavioral phenotype associated with DS. This psychometric validation is especially important for these measures as previous studies report expressive language artifacts in assessments of social cognition [21].

### Present Study

The current study aimed to evaluate social cognition and social behavior measures in children and adolescents with DS. Evaluated measures included the Developmental Neuropsychological Assessment, Second Edition (NEPSY-II) social cognition subtests and the SRS-2. The first aim of the study was to quantify the number of participants with DS who were able to obtain scores on the measures (i.e., feasibility). Score distributions were also examined to determine if there were floor or ceiling effects. The second aim evaluated test-retest reliability, practice effects, convergent validity, and associations with broader developmental domains (age, cognition, and language). Lastly, additional investigation was completed to determine what ages and cognitive levels were appropriate for administration of any subtest with low feasibility. Psychometric evaluation of social cognition and behavior measures will improve the quality of measurement in DS research and inform future clinical trials.

## 2. Materials and Methods

### 2.1. Participants

Participants were 6- to 17-year-old children and adolescents with DS (*n* = 73; M chronological age = 12.67, SD = 3.16). Average IQ was 48.70, SD = 4.76, and deviation scores were used for all analyses (M = 33.79, SD = 13.75; described below) [32]. There was an approximately equal ratio of males and females (54.8% male). Most participants were White (87.7%) and non-Hispanic (93.2%). Two parents reported that their child had ASD. Data from the participants in this study have been used in other manuscripts focused on the assessment of working memory outcome measures [33] and the association between executive function and adaptive skills [34].

### 2.2. Procedure

All study procedures were approved by the Streamlined, Multisite, Accelerated Resources for Trials (SMART) IRB platform at Cincinnati Children’s Hospital Medical Center (2018-0253, approved 23 April 2018), and informed consent was obtained for each subject before they participated. To be eligible for the study, participants were required to have a diagnosis of DS, have English as their primary language, and have an estimated nonverbal cognitive level of approximately three or older, per parent report, to support completion of at least a portion of the study procedures. Participants were recruited through medical clinics and DS associations at two sites. After being enrolled in the study, participants completed two visits, two weeks apart, as part of a broader longitudinal study on cognitive measurement in DS. To be included in analyses, participants were required to complete study measures at both time points.

### 2.3. Measures

#### 2.3.1. Overall Cognitive Ability

Stanford-Binet, Fifth Edition (SB-5) [35]. Overall IQ was measured using the abbreviated battery IQ (ABIQ) to describe the cognitive abilities of the sample and examine associations with social measures. The SB-5 is a standardized measure of cognition and the ABIQ includes one nonverbal (Fluid Reasoning) and one verbal (Knowledge) subtest. The ABIQ is strongly correlated with the full-scale IQ in samples of children with a neurodevelopmental disorder [36], and reliability is also high for the ABIQ (*r* = 0.85–0.96) [35]. Deviation scores were used in this study to eliminate floor effects (deviation scoring procedures described in [32]). The ABIQ deviation scores are an estimate of the full-scale deviation scores. Negative scores are possible using deviation scoring and represent raw scores that for a participant’s age are more than 3.33 standard deviations below the mean [32].

#### 2.3.2. Language

Expressive Vocabulary Test, Third Edition (EVT-3) [37]. The EVT-3 is an expressive vocabulary measure and is designed for individuals 2.5–90+ years old. Participants were shown a picture and asked to label the picture or provide a synonym using a one-word response. Standard scores were used for all analyses. Three participants were unable to complete the measure because of low verbal ability.

Peabody Picture Vocabulary Test, Fifth Edition (PPVT-5) [38]. The PPVT-5 is a receptive vocabulary measure and is designed for individuals 2.5–90+ years old. Participants were shown four response options and were required to select the picture that was compatible with the word provided by the examiner. Standard scores were used for all analyses.

#### 2.3.3. Social Cognition

Developmental Neuropsychological Assessment, Second Edition (NEPSY-II) [30]. The Theory of Mind and Affect Recognition subtests are both included in the NEPSY-II Social Perception domain and were selected to assess social cognition. Both subtests are designed for children as young as three years old and norming for the NEPSY-II included a variety of special group studies and small samples of children from clinical populations (e.g., intellectual disability, ADHD, and ASD). In the clinical sample, the correlation between Theory of Mind and Affect Recognition subtests is moderate (*r* = 0.53) and expected, considering the different abilities tested within the broader domain of social cognition.

##### NEPSY-II Theory of Mind

This subtest has verbal and nonverbal components and is designed to measure participants’ understanding of intention, deception, belief, emotion, and pretending. The perception of others’ thoughts, ideas, and feelings is also assessed. In the verbal portion of the task, the participant listens to scenarios or is shown images. The examiner asks a question about the point of view from a character in the presented information. In the nonverbal portion of the task (i.e., contextual task), the participant is presented with pictures of a social context and required to select the answer from four options that represents the correct affect of one of the persons pictured. NEPSY-II Theory of Mind test-retest reliability is high (*r =* 0.84). Because standard scores are only available up to 6:11, percentile ranks were used in addition to raw scores for measure description. Percentile ranks are unavailable for the total score for children 6–6:11 and therefore only verbal score percentile rank is reported for the 6-year-old participants. Verbal and total scores percentile rank are both reported for children 7–17 years old.

##### NEPSY-II Affect Recognition

This subtest is a nonverbal measure of the participant’s ability to identify emotions of children in photographs. Types of emotions presented include happy, sad, anger, fear, disgust, and neutral. The subtest has four subsets of tasks that vary in instruction and involve: (1) stating if two faces have the same affect, (2) selecting two faces with the same affect, (3) selecting the face that matches the affect of the face at the top of the page, and (4) choosing two faces from memory that match the affect of a previously shown face. In the third trial type, there are two items presented on each page, and the item not being administered is covered by the examiner to reduce distraction. The Affect Recognition subtest demonstrates adequate to good test-retest reliability (*r* = 0.46–0.66). Raw and standard scores were used for analyses.

#### 2.3.4. Social Behavior

Social Responsiveness Scale, Second Edition (SRS-2) [18]. The SRS-2 measures challenges with social interactions and communication. There are five subscales of the SRS-2 including Social Awareness, Social Cognition, Social Communication, Social Motivation, and Restricted Interests/Repetitive Behavior that produce a total score. The current study used parent report on the School-Age Form (ages 4–18). Parents were asked to rate their child’s behavior over the last six months. Internal consistency correlations for the SRS-2 are high in the publisher’s norming sample, which includes children with and without ASD (α = 0.95–0.97) [18]. Similarly, high internal consistency has also been reported in smaller samples of children with DS (α = 0.94–0.96) [19]. SRS-2 T-scores have a mean of 50 and a standard deviation of 10. Because both raw scores and T-scores had comparable results and there were no problems with score distributions identified, T-scores were used for all analyses.

### 2.4. Analysis Plan

To support Aim 1, the feasibility was assessed for measures of social cognition and social behavior administered to, or regarding, children and adolescents with DS. Feasibility was specified as the percentage of participants who provided responses at Time 1 and Time 2. Feasibility criteria were set a priori and ≥80% was the selected parameter for acceptable feasibility for use of these measures in DS research. This selection was informed by previous work on the psychometrics of cognitive measurements in intellectual disability and DS [33,39]. Examiners recorded reasons for noncompletion, which consisted of not understanding the task, behavioral noncompliance, and verbal refusal. Noncompletion of the parent-report measure was from missing questions (i.e., did not complete both sides of paper form) or failure to return the questionnaire. Range of scores, skewness, and kurtosis were also examined to determine the normality of the score distributions and to evaluate if there were floor effects for raw or standard scores. Acceptable values for skewness were between −1 and 1 and were between −2 and 2 for kurtosis. Participants who completed the measure with the lowest possible score, and those who were unable to complete the measure at Time 1, were both included in the estimate of floor effects. Floor effects < 20% were considered appropriate for research.

To support Aim 2, further psychometric evaluation (test-retest reliability, practice effects, and validity) was completed over the two-week testing interval. Test-retest reliability was assessed using intraclass correlation coefficients (ICC). Descriptive categories for ICCs are poor (<0.50), moderate (0.50−0.74), good (0.75−0.90), or excellent (>0.90) [33,40], and a priori good or excellent classifications were deemed suitable. Paired samples *t*-tests were used to assess practice effects. Practice effects were presumed if scores at the two testing visits had a significance value less than 0.05 and Cohen’s *d* effect size greater than 0.20. Convergent validity across a selection of measures (NEPSY-II subtests and SRS-2 Social Awareness and Social Cognition) and associations among all social measures was determined using bivariate Pearson correlations. Correlation coefficients ≥0.50 were deemed as acceptable for convergent validity. Associations with broader developmental domains (age, cognition, and language) were also evaluated, and significant correlations were expected.

The third aim of the study investigated measures with low feasibility using post hoc sensitivity and specificity analyses. Sensitivity probabilities estimate the likelihood that a participant with specific characteristics will be able to complete the measure. Specificity probabilities estimate the likelihood that a participant not included in the specified characteristics will be unable to complete the measure. These analyses were completed for any measure that did not meet study feasibility criteria, and suggestions for age and cognitive ability of participants for future administration were established (as per [33]). Benchmarks for sensitivity and specificity probabilities were selected based on age (8 and 10 years) and cognitive ability (ABIQ deviation scores ≥20, ≥30, ≥40, and ≥50). Lower bounds of chronological age in previous clinical trials in DS informed benchmark selection [41].

## 3. Results

### 3.1. Aim 1: Feasibility and Floor Effects

Feasibility and floor effect indices for raw scores, percentile ranks, and/or standard scores (as appropriate) of the NEPSY-II Theory of Mind, NEPSY-II Affect Recognition, and SRS-2 are presented in Table 1. Two of the three measures evaluated in this study met the a priori criterion for feasibility: the NEPSY-II Theory of Mind (86.3–87.7%) and the SRS-2 (87.7%). NEPSY-II Affect Recognition fell below acceptable criterion for feasibility (71.2%) and therefore was investigated for Aim 3 as part of the post hoc analysis for low feasibility measures. Reasons for missing the NEPSY-II Affect Recognition subtest included not understanding the task (17.8%), behavioral noncompliance (3.4%), and verbal refusal (0.7%). A small portion of participants only completed the subtest at one time point (6.9%). Additionally, 15.4% of participants who completed the measure were described as exhibiting “acquiescence”, defined as selecting responses without considering each response option. Floor effects followed the same pattern for the NEPSY-II raw scores and SRS-2 T-scores, with acceptable levels of floor effects for the NEPSY-II Theory of Mind (15.1–19.2%) and the SRS-2 (12.3%), and unacceptable levels for the NEPSY-II Affect Recognition (28.8%). Floor effects for percentile rank on the NEPSY-II Theory of Mind and standard scores on the NEPSY-II Affect Recognition were both below a priori criteria. Specifically, of the participants who could complete the measures, 95% had the lowest percentile rank (<2%) on the NEPSY-II Theory of Mind Verbal, 97% had the lowest percentile rank (<2%) on the NEPSY-II Theory of Mind Total, and 39% had the lowest standard score (1) on the NEPSY-II Affect Recognition. Table 1 presents floor effects that include those with the lowest score on each measure and those who were unable to complete the task.

### 3.2. Aim 2: Test-Retest Reliability, Practice Effects, and Validity

#### 3.2.1. Test-Retest Reliability and Practice Effects

Overall test-retest reliability ranged from poor to excellent on the evaluated measures (Table 2). Raw scores for the NEPSY-II Theory of Mind verbal and total scores were in the moderate range for test-retest reliability, falling below a priori criterion for this study. The NEPSY-II Affect Recognition had poor test-retest reliability, again below acceptable criterion. The SRS-2 had moderate to excellent test-retest reliability and all ICCs were 0.70 or greater. The majority were above 0.75 a priori criterion, therefore demonstrating stable test-retest reliability across the two-week testing interval. There were no practice effects on any of the measures evaluated in this study (see Table 2).

#### 3.2.2. Convergent Validity and Associations among Social Measures

Convergent validity was assessed for a selection of measures (NEPSY-II subtests and SRS-2 Social Awareness and Social Cognition), and correlations among all social measures were examined (Table 3). Associations between NEPSY-II Theory of Mind and Affect Recognition were below the acceptable criterion of *r* > 0.50; however, the correlation coefficients of 0.43–0.51 were similar to data on the relation between the two measures reported by the publisher (*r* = 0.53) [30]. Significant associations were also found between SRS-2 Social Awareness and SRS-2 Social Cognition. However, there were no significant associations between the NEPSY-II subtests and the SRS-2 Social Awareness or Social Cognition. Although not all subdomains of the SRS-2 are theoretically aligned with the NEPSY-II direct assessments of social cognition, it is noteworthy that no SRS-2 subscales were correlated with NEPSY-II social cognition subtests. Within the SRS-2, all subscales were positively correlated, and the strength of many of the associations were strong (0.34–0.94).

#### 3.2.3. Associated Developmental Domains

Significant positive correlations were observed between NEPSY-II subtests and ABIQ deviation scores, EVT-3 standard scores, and PPVT-5 standard scores (Table 2). For the NEPSY-II Theory of Mind, associations with all three cognition and language measures were moderately strong (0.41–0.51). The NEPSY-II Affect Recognition subtest was also positively correlated with ABIQ deviation scores and EVT-3 standard scores; however, no association was found with PPVT-5 standard scores. The SRS-2 had modest correlations with ABIQ deviation scores in the expected direction, such that more social behavior challenges were associated with lower ABIQ. In most cases, there was no significant correlation between the SRS-2 and PPVT-5 or EVT-3 standard scores. The majority of the measures were not associated with chronological age, with the exception of the NEPSY-II Affect Recognition Total raw score, which was positively correlated with age (*r* = 0.32).

### 3.3. Aim 3: Assessments with Low Feasibility

**Figure 1:**
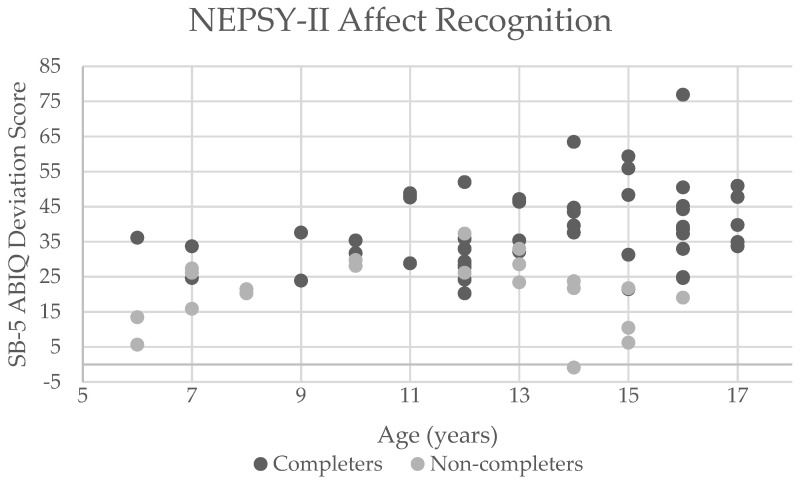
Chronological age in years and Stanford-Binet, Fifth Edition abbreviated battery IQ deviation scores of completers and non-completers for NEPSY-II Affect Recognition. Negative score represents a raw score more than 3.33 standard deviations below the mean that for the participant’s age [32].

The NEPSY-II Affect Recognition was the only measure to fall below the feasibility threshold in this study. To better understand the subset of the population within DS that this measure would be appropriate for, sensitivity and specificity calculations were completed (Table 4). Less restrictive guidelines (i.e., ABIQ deviation ≥ 20 or 30) provided higher sensitivity, indicating that completers of the measure were correctly identified. As guidelines become more restrictive (i.e., ABIQ deviation ≥ 40 or 50), sensitivity decreased, and not all participants who could complete the task were identified using the more limiting benchmarks. More restrictive ABIQ also led to higher specificity, indicating that those who were *not* able to complete the measure were correctly identified when using those more restrictive benchmarks. Chronological ages examined (8 and 10 years) revealed minimal differences between sensitivity and specificity probabilities. Figure 1 illustrates the chronological age and ABIQ deviation scores of both completers and non-completers for the NEPSY-II Affect Recognition in our sample.

## 4. Discussion

This study evaluated the psychometric properties of two clinical assessments of social cognition and one social behavior parent questionnaire (summarized in Table 5). Both direct assessments of social cognition and parent-report of social behavior met criteria for a portion of the psychometric indices evaluated. Associations with cognition and language abilities emphasize how these social cognition and behavior measures relate to broader developmental domains. Additionally, the relations among measures show a clear pattern of correlation within NEPSY-II subtests and within SRS-2 subdomains, but no correlations were found across the direct assessments and parent-report measures. The SRS-2 demonstrated the strongest psychometric properties, with high feasibility, moderate to excellent test-retest reliability, and no practice effects, suggesting this measure could be appropriate for use in clinical trials involving youth with DS. The NEPSY-II Theory of Mind subtest raw scores also demonstrated good psychometrics; however, percentile rank floor effects indicate this measure is not suitable for this population. Feasibility for the NEPSY-II Affect Recognition was problematic and this measure may only be appropriate for certain IQ ranges of children and adolescents with DS.

### 4.1. Feasibility and Floor Effects

The NEPSY-II Theory of Mind and SRS-2 both met a priori feasibility criteria and over 85% of participants obtained scores on these measures. Although feasibility was adequate for these measures, percentile ranks were at the floor for the NEPSY-II Theory of Mind. Therefore, we recommend raw scores for use in future work utilizing this measure. Despite relative challenges with theory of mind in DS [14,15], it is encouraging that raw scores were able to capture a range of scores on this measure; however, percentile rank floor effects show that this measure does not discriminate performance between subjects in the sample using published norms. Further, there were minimal differences between raw scores and T-scores on the SRS-2, and the use of T-scores is appropriate for this measure of social behavior. High feasibility of the SRS-2 reinforces the suitability of this tool for the measurement of social behavior in individuals with DS [5,19]. Feasibility was below a priori criterion for the NEPSY-II Affect Recognition and floor effects were observed for both raw and standard scores and most problematic for standard scores. Low feasibility and standard scores on this measure may be, in part, due to difficulties individuals with DS have with recognizing emotional expressions in others [16,17]. Difficulty understanding the task was the greatest reason for noncompletion and assessments of affect recognition with simpler instructions and task demands may be needed for this population. Recommendations for future use of the NEPSY-II Affect Recognition in DS are provided in the discussion of low feasibility measures below.

### 4.2. Test-Retest Reliability, Practice Effects, and Validity

There were mixed results regarding the reliability and validity of the evaluated measures. Test-retest reliability was strongest for the SRS-2 subscales, providing evidence for consistent reports of social behavior by caregivers. Social Awareness had the lowest ICC for the SRS-2 (0.70), which was in the moderate range, but close to the “good” category (0.75). Although moderate test-retest reliability was found for NEPSY-II Theory of Mind raw scores, NEPSY-II Affect Recognition raw scores demonstrated poor reliability. Inconsistent scores between the two-week test-retest interval on the NEPSY-II Affect Recognition indicate that children may be guessing or acquiescing with their responses. For all evaluated measures, NEPSY-II subtests and SRS-2, practice effects were negligible. The lack of improvement at the second study time point suggests the measures were stable with multiple administrations over a relatively short period.

Investigation of convergent validity resulted in no association between parent reports of social cognition/awareness and direct assessments of social cognition. These different test modalities may be tapping different constructs or skills, as there are clear differences in laboratory-based assessments compared with parent-report measures. Therefore, while the NEPSY-II Theory of Mind shows some good psychometric properties, we need to consider what it is measuring. It may be the case that standardized clinical assessments of theory of mind do not represent parental perceptions of a child’s daily abilities in social awareness and understanding. The NEPSY-II Theory of Mind may also have poor ecological validity. Further, the NEPSY-II Theory of Mind is moderately correlated with receptive and expressive language, and overall language abilities may be confounding performance, as has occurred in previous studies [21]. Another plausible interpretation is that social abilities reported by parents are truly different skills than those assessed in the laboratory. Although there were no associations between the NEPSY-II and SRS-2, there were significant associations among the SRS-2 subscales, which parallels previous significant correlations reported among SRS subscales in DS [19].

Associations with broader developmental domains varied significantly across measures. First, both NEPSY-II subtests and SRS-2 subscales had significant correlations with cognitive ability, in the expected directions, such that higher cognitive abilities were associated with better social cognition and fewer social behavior challenges. The associations between the SRS-2 subscales and cognitive abilities have not been consistently found in previous investigations between SRS-2 and nonverbal IQ in DS [5,19], but this study does replicate a moderate association found between cognition and SRS Total T-scores [19], despite using different IQ measures. Correlations between NEPSY-II subtests and ABIQ were markedly stronger than comparisons between the SRS-2 and ABIQ. This reinforces the idea that direct assessment may be tapping similar skills that are fundamentally different from the behaviors and performance observed by parents in the home environment. Both NEPSY-II subtests were positively correlated with the expressive language measures, but only the NEPSY-II Theory of Mind subtest was associated with receptive language. This highlights the receptive language demands of the NEPSY-II Theory of Mind that are required to complete the measure. SRS-2 Social Awareness was the only subscale that was associated with receptive language, which deviates from previous reports of a significant association between all SRS subscales and receptive vocabulary [19]. This study also replicated previous reports of no correlation between the SRS-2 subscales and expressive language [5]. Finally, associations with chronological age were minimal and corroborate previous reports of a lack of association with the SRS [5,19], suggesting that developmental level is a better indicator of social cognition and behavior than age.

### 4.3. Assessments with Low Feasibility

Because the NEPSY-II Affect Recognition had feasibility that fell below a priori criterion, follow-up post hoc sensitivity and specificity analyses were used to describe who the measure is appropriate for within the sample of children and adolescents with DS. There was a clear pattern that less restrictive guidelines led to more sensitivity, correctly identifying any participant who could complete the task. More restrictive guidelines led to more precision and greater confidence that those in the high IQ ranges could complete the measure (i.e., specificity). It is ideal to have a balance of both high sensitivity and specificity to avoid missing participants who could complete the task but also to be administering a task appropriate for the individuals in a study or clinical trial. The current study’s benchmark of ABIQ deviation scores ≥ 30 had the greatest balance between sensitivity and specificity probabilities and would be appropriate for inclusion/exclusion criteria if the NEPSY-II Affect Recognition were a required measure for a testing battery. However, there are limitations to this benchmark, as there were some participants below the ABIQ deviation score of 30 who were able to complete the measure.

### 4.4. Limitations and Future Directions

The current study provides essential information about the psychometric properties of social cognition and social behavior measures in DS, but it also has limitations. First, the rates of ASD in our study sample were lower than what has been reported in other studies examining the prevalence of ASD in DS, and additional work is needed to determine if these measures are appropriate for participants with DS and co-occurring ASD. There is also a need for a longer follow-up period to determine how these measures assess social constructs over 6 months or a year, to match the study design of a clinical trial. Examining the psychometrics of the measures in groups of children within narrower age ranges will also be an important step for future research. Additionally, social behavior was only measured using parent-report, and while it is valuable to understand the comparison between direct assessment and parent-report, this study did not include any direct assessments of social behavior. Because the NEPSY-II laboratory-based assessments were not correlated with the SRS-2 Social Cognition and Social Awareness, additional work is also needed to determine the generalizability of NEPSY-II subtests to real-world contexts. Finally, because few standardized clinical assessments focus on social cognition, further examination of a greater variety of social cognition laboratory-based measures is needed to ensure that the measures appropriate for the general population [29,31] are also suitable for individuals with DS. This future work would help to identify additional alternatives for measuring social cognition and social behavior in DS.

## 5. Conclusions

Findings from this study add to the list of standardized measures that may be used in clinical trials with children and adolescents with DS. The SRS-2 T-scores had normal distributions, good feasibility, moderate to excellent test-retest reliability, and no practice effects, and therefore this measure could be suitable for use in clinical trials. Although the NEPSY-II Theory of Mind raw scores were psychometrically sound, the measure was problematic overall, considering the percentile rank floor effects and lack of evidence for ecological validity. Researchers should also use caution when using NEPSY-II Affect Recognition, as feasibility was problematic in the current study. We recommend referencing the sensitivity and specificity benchmarks when using this measure to guide decisions about inclusion/exclusion criteria in future studies with this population. The psychometric evaluation of social cognition and social behavior measures supports the NIH working group initiative of determining appropriate outcome measures for individuals with DS [27] and will contribute to the success of future clinical trials in DS.

## Figures and Tables

**Table 1:** Social cognition performance and feasibility at Time 1, *n* = 73.

	Min	Max	Median	Skew	Kurtosis	Feasibility *n* (%)	*n* at Floor ^a^
**NEPSY-II subtests**							
ToM Verbal Raw Score	0	18	5.5	0.74	0.15	64 (87.7%)	14/73
ToM Verbal Percentile Rank ^b^	<2	11–25	-	-	-		70/73
ToM Total Raw Score	0	22	7	0.64	-0.01	63 (86.3%)	11/73
ToM Total Percentile Rank ^b^	<2	2–5	-	-	-		69/70 ^c^
AR Total Raw Score	1	26	13	−0.20	−0.57	52 (71.2%)	21/73
AR Total Standard Score	1	8	2	0.94	−0.32		41/73
**SRS-2 T-scores**							
Total	42	86	60.5	0.54	0.39	64 (87.7%)	9/73
Social Awareness	37	79	60	−0.18	−0.37		
Social Cognition	43	82	65	−0.08	−0.68		
Social Communication	41	90	60	0.83	1.35		
Social Motivation	40	90	54	1.10	1.64		
RRBI	45	90	57	0.65	−0.40		

^a^*n* at floor includes children who got the lowest score on the measure and those who could not complete the task; ^b^ Some descriptive statistics not reported for percentile ranks; ^c^ NEPSY-II Theory of Mind Total score percentile rank is not normed for 6-year-olds (*n* = 3); ToM = Theory of Mind; AR = Affect Recognition; SRS-2 = Social Responsiveness Scale, Second Edition; RRBI = Restricted, Repetitive Behaviors and Interests.

**Table 2:** Examination of practice effects, test-retest reliability, and correlations with broader developmental domains.

	Time 1 Mean (SD)	Time 2 Mean (SD)	*t*	*p*	*d*	ICC	Age	ABIQ ^a^	EVT-3 SS	PPVT-5 SS
**NEPSY-II subtests**										
ToM Verbal Raw Score	5.63 (4.24)	5.03 (3.85)	1.37	0.18	0.15	0.63	0.13	0.50 ***	0.42 ***	0.41 **
ToM Total Raw Score	8.19 (4.77)	7.92 (4.34)	0.57	0.57	0.06	0.66	0.15	0.51 ***	0.47 ***	0.44 ***
AR Total Raw Score	13.48 (6.80)	13.23 (6.76)	0.25	0.81	0.04	0.41	0.32 *	0.59 ***	0.39 **	0.25
**SRS-2 T-scores**										
Total	61.28 (9.67)	61.67 (10.87)	−0.78	0.44	0.04	0.92	0.08	−0.31 *	−0.08	−0.21
Social Awareness	59.47 (9.04)	59.72 (9.06)	−0.03	0.78	0.03	0.70	−0.08	−0.32 *	−0.19	−0.25 *
Social Cognition	63.64 (9.24)	64.34 (9.99)	−1.01	0.32	0.07	0.83	−0.02	−0.32 *	−0.15	−0.22
Social Communication	61.02 (9.46)	61.86 (11.34)	−1.24	0.22	0.08	0.86	0.06	−0.29 *	−0.05	−0.17
Social Motivation	54.25 (10.38)	53.86 (11.64)	0.59	0.56	0.04	0.89	0.11	−0.11	−0.19	−0.01
RRBI	60.16 (10.84)	60.36 (11.64)	−0.34	0.74	0.02	0.91	0.15	−0.27 *	−0.17	−0.24

* *p* < 0.05, ** *p* < 0.01, *** *p* < 0.001; ^a^ Stanford-Binet, Fifth Edition Deviation Scores; ABIQ = Abbreviated Intelligence Quotient; PPVT-5 = Peabody Picture Vocabulary Test, Fifth Edition; EVT-3 = Expressive Vocabulary Test, Third Edition; SS = Standard Score; SRS-2 = Social Responsiveness Scale, Second Edition; ICC = intraclass correlation coefficients; ToM = Theory of Mind; AR = Affect Recognition; RRBI = Restricted, Repetitive Behaviors and Interests; *d* = Cohen’s *d*.

**Table 3:** Bivariate Pearson correlations to assess convergent validity and associations among social measures at Time 1.

	1	2	3	4	5	6	7	8
1. Theory of Mind Verbal Raw Score								
2. Theory of Mind Total Raw Score	0.96 ***							
3. Affect Recognition Total Raw Score	0.43 **	0.51 **						
4. SRS-2 Social Awareness	−0.14	−0.14	−0.21					
5. SRS-2 Social Cognition	−0.15	−0.15	−0.18	0.76 ***				
6. SRS-2 Social Communication	−0.05	−0.08	−0.04	0.69 ***	0.76 ***			
7. SRS-2 Social Motivation	0.09	0.07	0.11	0.34 **	0.56 ***	0.71 ***		
8. SRS-2 RRBI	−0.18	−0.14	−0.11	0.54 ***	0.60 ***	0.69 ***	0.64 ***	
9. SRS-2 Total	−0.10	−0.10	−0.08	0.74 ***	0.85 ***	0.94 ***	0.80 ***	0.83 ***

** p < 0.01; *** *p* < 0.001; SRS−2 = Social Responsiveness Scale, Second Edition; RRBI = Restricted, Repetitive Behaviors and Interests.

**Table 4:** Post hoc sensitivity and specificity for the measure below feasibility criteria.

	NEPSY-II Affect Recognition			
	Sensitivity		Specificity	
	Age 8	Age 10	Age 8	Age 10
No ABIQ ^a^ Restriction	94.2%	88.5%	23.8%	33.3%
ABIQ ^a^ ≥ 20	94.0%	88.2%	42.9%	52.4%
ABIQ ^a^ ≥ 30	74.0%	70.6%	90.5%	90.5%
ABIQ ^a^ ≥ 40	38.0%	38.0%	100%	100%
ABIQ ^a^ ≥ 50	14.0%	14.0%	100%	100%

ABIQ ^a^ = Stanford-Binet, Fifth Edition abbreviated battery IQ deviation score.

**Table 5:** Summary of social cognition and behavior measures assessed on a priori criteria.

	Minimal Floor Effects	Feasibility	Test-Retest	Negligible Practice Effects
**NEPSY-II**				
Theory of Mind Verbal Raw Score	+	+	−	+
Theory of Mind Total Raw Score	+	+	−	+
Affect Recognition Total Raw Score	−	−	−	+
**SRS-2 T scores**				
Social Awareness	+	+	−	+
Social Cognition	+	+	+	+
Social Communication	+	+	+	+
Social Motivation	+	+	+	+
RRBI	+	+	+	+
Total	+	+	+	+

+ indicates study criterion met: <20% floor effects, ≥80% feasibility, ≥0.75 test-retest ICC, small and non-significant practice effects; − indicates study criterion not met: ≥20% floor effects, <80% feasibility, <0.75 test-retest ICC, medium/large and significant practice effects; RRBI = Restricted, Repetitive Behaviors and Interests.

## Data Availability

The data that support the findings of this study are available from the corresponding author upon reasonable request.
